# A Scoring System for Outpatient Orthopedist to Preliminarily Distinguish Spinal Metastasis from Spinal Tuberculosis: A Retrospective Analysis of 141 Patients

**DOI:** 10.1155/2021/6640254

**Published:** 2021-05-29

**Authors:** Xing Du, Yuxiao She, Yunsheng Ou, Yong Zhu, Wei Luo, Dianming Jiang

**Affiliations:** Department of Orthopedics, The First Affiliated Hospital of Chongqing Medical University, Chongqing 400016, China

## Abstract

**Objective:**

Spinal tuberculosis (TB) misdiagnosed of spinal metastasis was not rarely reported, especially in outpatients department. This study was aimed to establish an outpatient scoring system to preliminarily distinguish spinal metastasis from spinal TB.

**Methods:**

We retrospectively reviewed consecutive 141 patients with a pathological diagnosis of spinal metastasis (82 cases) or spinal TB (59 cases) in our hospital from January 2017 to June 2018. The following clinical characteristics which can be obtained by outpatient orthopedist were recorded and analyzed: age, gender, malignant tumor history, erythrocyte sedimentation rate (ESR), C-reactive protein (CRP), and imaging features including distribution characteristics of vertebral lesions, subligamentous spread, paravertebral or psoas abscess, involved vertebral element, intervertebral disc, and sequestra formation. The prevalence of clinical characteristics in spinal metastasis was evaluated, and the scoring system was established using logistic regression analysis. The performance of the scoring system was also prospectively validated.

**Results:**

The outpatient scoring system was based on five clinical characteristics confirmed as significant predictors of spinal metastasis, namely, malignant tumor history, subligamentous spread, posterior element lesions, preserved discs, and no sequestra formation. Spinal metastasis showed a significant higher score than spinal TB (8.17 points *vs.* 1.97 points, *t* = 18.621, *P* < 0.001), and the optimal cut-off value for the scoring system was 5 points. The sensitivity and specificity of the scoring system for predicting spinal metastasis were 97.85% and 88.33%, respectively, in the validation set.

**Conclusion:**

Spinal lesions with the score of 5 to 10 would be considered a diagnosis of spinal metastasis, while the score of 0 to 4 may be spinal TB. Because the scoring system is mainly based on the clinical characteristics that can be obtained by an outpatient orthopedist, it is suitable to be used as a diagnostic tool in the outpatient department.

## 1. Introduction

Spinal metastasis and spinal TB are both common spinal lesions [1, 2], but the treatment of them are quite different. Spinal metastasis is a malignant lesion and surgery might be an optimal therapy [3], while spinal TB is a benign disease and effective anti-TB chemotherapy is of great importance [4]. So distinguishing spinal metastasis from spinal TB is essential to reduce pain, prevent neurological disability, minimize spinal deformity, and improve prognosis [5, 6].

However, spinal metastasis and spinal TB show similar clinical manifestations and imaging features such as back pain, weakness, weight loss, vertebrae destruction, pathological fracture, kyphosis deformity, and even neurological dysfunction [7], so it is difficult to distinguish the two accurately, especially in the outpatient department because of the limited consultation time and examination condition [8]. Although biopsy has been proved as the gold standard to distinguish spinal metastasis from spinal TB [9], it cannot be conducted in the outpatient department, so in actual outpatient work, the diagnosis was mainly dependent on the combination of clinical findings and auxiliary examination [3, 10]. However, because not everyone shows the typical clinical characteristics of spinal metastasis or spinal TB, spinal TB misdiagnosed of spinal metastasis was not rarely reported, even during the hospitalization [11–14]. What was worse, incorrect outpatient diagnosis may give a negative effect on the patients' treatment choice [15, 16]. For example, misdiagnosis of spinal TB as spinal metastasis may result in patient's giving up of hospitalization, missing the best opportunity for treatment, and wasting medical resources, especially for the poor patients [17]. Therefore, it is important to establish a new method to improve the accuracy of distinguishing spinal metastasis from spinal TB in the outpatient department to help the outpatients receive optimal therapy.

In this study, we retrospectively analyzed the clinical characteristics of spinal metastasis and spinal TB and confirmed five characteristics which can be obtained by outpatient orthopedist as significant predictors of spinal metastasis and developed an outpatient scoring system. We also validated the performance of this scoring system and confirmed that it can improve the ability to distinguish spinal metastasis from spinal TB.

## 2. Materials and Methods

This study was approved by the Ethics Committee of the First Affiliated Hospital of Chongqing Medical University (2017-99). All of the participants provided their written informed consent to participate in this study. The work has been reported in line with the STARD criteria.

### 2.1. Patients Selection

We retrospectively reviewed the medical records of hospitalized patients diagnosed of spinal metastasis or spinal TB in our department from January 2017 to June 2018 to form the derivation set.

#### 2.1.1. Inclusion Criteria

(1) The medical records were complete, including the general information of the patient, preoperative laboratory examination, and imaging results (MRI and CT were both performed). (2) Patients who were preliminarily diagnosed with spinal metastasis or spinal TB according to clinical symptoms and results of auxiliary examinations before surgery. (3) Patients who underwent surgical treatment (including minimally invasive surgery or open surgery). (4) Lesion tissues were taken during the surgery, and postoperative pathological diagnosis was spinal metastasis or spinal TB

#### 2.1.2. Exclusion Criteria

(1) Patients with suspected spinal metastasis or spinal TB who were not been confirmed by pathological examination. (2) Patients with preliminary and pathological diagnosis of other diseases other than spinal metastasis or spinal TB. (3) Patients with a previous history of spinal metastasis or spinal TB.

### 2.2. Data Collection

Based on the results of previous studies and our experience, we included the possible predictors for differential diagnosis of spinal metastasis and spinal TB, which mainly included patients' general conditions, laboratory examination indexes, and imaging examination indexes.

**Figure 1:**
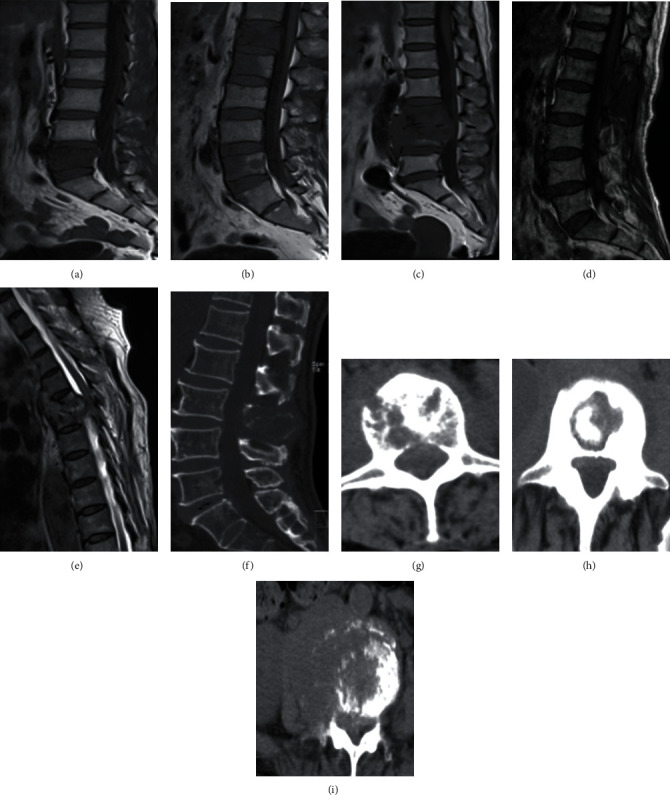
Included imaging features. (a) Isolated vertebral lesion. (b) Skipped vertebral lesions. (c) Contiguous vertebral lesions and subligamentous spread of abscess. (d) Preserved intervertebral disc. (e) Destroyed intervertebral disc and subligamentous spread of abscess. (f) Destroyed vertebral posterior elements. (g) Destroyed vertebral body. (h) Sequestra formation. (i) Paravertebral abscess.

1. General conditions: age, gender, and malignant tumor history
2. Laboratory examination indexes: ESR and CRP
3. Imaging examination indexes: two spinal surgeons with more than five years of experiences and who were blinded as to the patients' diagnosis independently reviewed all MRI and CT images and recorded the lesion characteristics (Figure 1): distribution characteristics of vertebral lesions (such as isolated, skipped or contiguous), subligamentous spread, paravertebral or psoas abscess, and involved vertebral elements (vertebral body or posterior elements such as lamina, pedicle, or spinous process); whether the intervertebral disc was destroyed or not and whether sequestra was formed. If there was any disagreement between the two surgeons, the consensus decision was made after a discussion with the third surgeon

### 2.3. Development of the Scoring System

Firstly, all the included patients were divided into two groups, namely, spinal metastasis or spinal TB according to their pathological diagnosis.

Secondly, we converted the continuous variables (age, ESR, and CRP) to dichotomous variables. The threshold values of continuous variables for predicting spinal metastasis were obtained using receiver operating characteristic (ROC) curves analysis.

Thirdly, univariate analysis was conducted on the general conditions, laboratory examination indexes, and imaging examination indexes of patients in the two groups. Based on the results of univariate analysis, the index with a *P* value less than 0.05 was considered a possible predictor for differential diagnosis between spinal metastasis and spinal TB.

Next, multivariate logistic regression analysis was performed for the indexes with *P* values less than 0.05 in univariate analysis. According to the results of multivariate logistic regression analysis, the indexes with *P* values less than 0.05 were considered the final predictors for differential diagnosis between spinal metastasis and spinal TB and, thus, determined as the items of the scoring system.

Then, we established the weighted score of each item based on the relative size of the *β*-coefficient according to the method reported by Kharbanda et al. [18] and Zhou et al. [19].

Finally, we made the appropriate cut-off points for the scoring system using ROC curves corresponding to the point on the curve nearest the upper left corner of the ROC graph.

### 2.4. Validation of the Scoring System

From July 2018 to December 2020, we prospectively included outpatients to validate the accuracy of the scoring system. The following criteria were used to determine whether an outpatient should be prospectively included in the validation set.

Inclusion criteria: (1) the outpatients' general information and preoperative imaging examination results were all accessible for the outpatient orthopedist. (2) According to the scoring system, outpatients were preliminarily diagnosed with spinal metastasis or spinal TB. (3) Outpatients were willing to be hospitalized for surgical treatment.

Exclusion criteria: (1) outpatients with a preliminary diagnosis of other diseases other than spinal metastasis or spinal TB. (2) Outpatients had no enough preoperative data and the outpatient orthopedist could hardly make a diagnosis based on the scoring system.

After surgery, the final pathological diagnosis of the included patients was recorded. The accuracy of the scoring system was evaluated by comparing the consistency between the preliminary diagnosis and the final pathological diagnosis.

### 2.5. Statistical Analysis

Both the threshold values for continuous variables and the appropriate cut-off points for the scoring system were determined by the ROC curves analysis. The prevalences of included clinical characteristics were evaluated by calculating the sensitivity and specificity for each factor. The clinical characteristics were also subjected to univariate logistic regression analysis, and the significant factors were evaluated by multivariate logistic regression analysis. The items of the scoring system were determined by multivariate logistic regression, and the weighted score of each item was based on the relative size of the *β*-coefficient. *P* < 0.05 was set of statistical significance. The SPSS version 10.0 software was used for statistical analysis.

## 3. Results

### 3.1. Patients Population

Finally, a total of 141 patients were included in the derivation set, including 82 cases of spinal metastasis and 59 cases of spinal TB, according to the pathological examination. The primary lesions of 82 spinal metastases were as follows: 28 cases with lung cancer, 13 cases with breast cancer, 10 cases with prostate cancer, 7 cases with liver cancer, 6 cases with cervical cancer, 5 cases with kidney cancer, 4 cases with gastrointestinal cancer, 3 cases with ovary cancer, 2 cases with bladder cancer, 2 cases with thyroid gland cancer, 1 case with nasopharynx cancer, and 1 case with parotid gland cancer. 48 men and 34 women were diagnosed with spinal metastasis, and 36 men and 23 women were spinal TB. The mean ages of spinal metastasis and spinal TB groups were 54.09 ± 15.71 years and 48.25 ± 15.65 years, respectively.

### 3.2. Derivation of the Scoring System: Univariate Analysis

**Figure 2:**
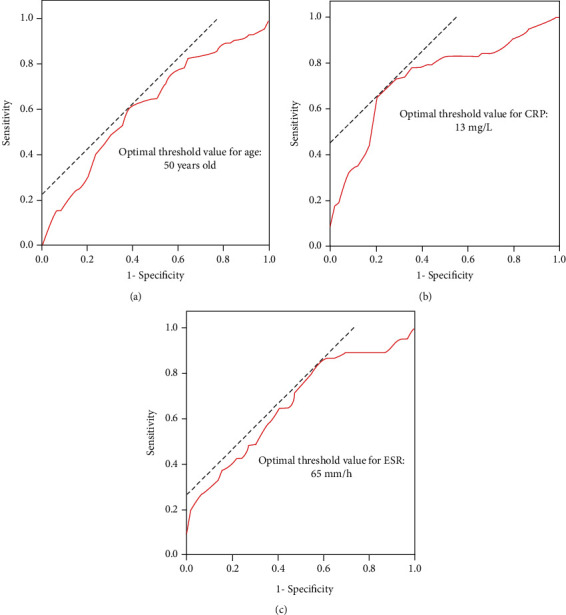
Threshold values for continuous variables. ROC curve analysis showed that the optimal threshold values for age (a), CRP (b), and ESR (c) were 50 years old, 65 mm/h, and 13 mg/L, respectively.

ROC curve analysis showed that (1) the best threshold value for age was 50 years old, the area under curve (AUC) was 0.614 (95% CI: 0.520-0.728, *P* = 0.021), and the diagnostic accuracy was low (Figure 2(a)). (2) The best threshold value for CRP was 13 mg/L, the AUC was 0.739 (95% CI: 0.656-0.823, *P* < 0.001), and the diagnostic accuracy was moderate (Figure 2(b)). (3) The best threshold value for ESR was 65 mm/h, the AUC was 0.670 (95% CI: 0.581-0.759, *P* = 0.001), and the diagnostic accuracy was low (Figure 2(c)). These continuous variables were converted to categorical variables on the basis of these threshold value. The relationship between each categorical variable and spinal metastasis was evaluated with chi-square analysis (Table 1).

### 3.3. Development of the Scoring System

Multivariate logistic regression analysis was carried out on the significant findings in univariate analysis and showed five clinical characteristics, namely, malignant tumor history, no subligamentous spread, and posterior elements of vertebrae were involved, and intervertebral disc was normal were significant predictors of spinal metastasis (Table 2).

In order to distinguish spinal metastasis from spinal TB, we developed a scoring system based on five clinical characteristics that were conformed significant predictors of spinal metastasis. The variables with significant predictive value for spinal metastasis were given the weighted scores according to the relative value of the *β*-coefficient in multivariate logistic regression analysis: malignant tumor history, no subligamentous spread, vertebral posterior elements destroyed, no sequestra formation, and preserved intervertebral discs were weighted as 2 points, 1 point, 2 points, 3 points, and 2 points, respectively. The score was then calculated by determining the total number of points, ranging from 0 to 10 (Table 3).

**Figure 3:**
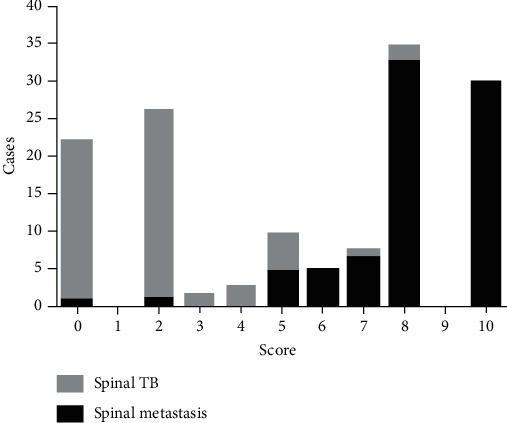
Histogram distribution of spinal metastasis and spinal TB for each score.

**Figure 4:**
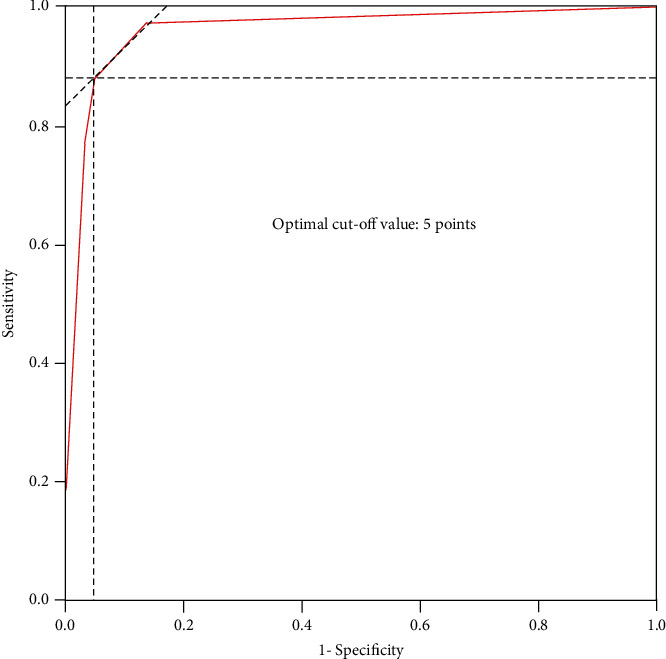
ROC curve analysis of the outpatient scoring system. The optimal cut-off point based on the ROC curve analysis of scores was 5 points.

A histogram distribution of the score values was shown in Figure 3. Remarkably, spinal metastasis showed a significant higher score than spinal TB (8.17 points *vs.* 1.97 points, *t* = 18.621, *P* < 0.001). The optimal cut-off value of the scoring system was 5 points, and the AUG was 0.965 (95% CI: 0.935-0.996, *P* < 0.001) (Figure 4).

### 3.4. Validation of the Scoring System

Finally, a total of 153 patients were prospectively included in the validation set, including 98 cases of spinal metastasis and 55 cases of spinal TB according to the pathological examination.

Comparison of the performance of the score system on derivation set and validation set was shown in Table 4. Based on the cut-off value of 5 points, the sensitivity and specificity of the score for predicting spinal metastasis were 97.56% and 86.44%, respectively, in derivation set and 97.85% and 88.33% in validation set.

**Figure 5:**
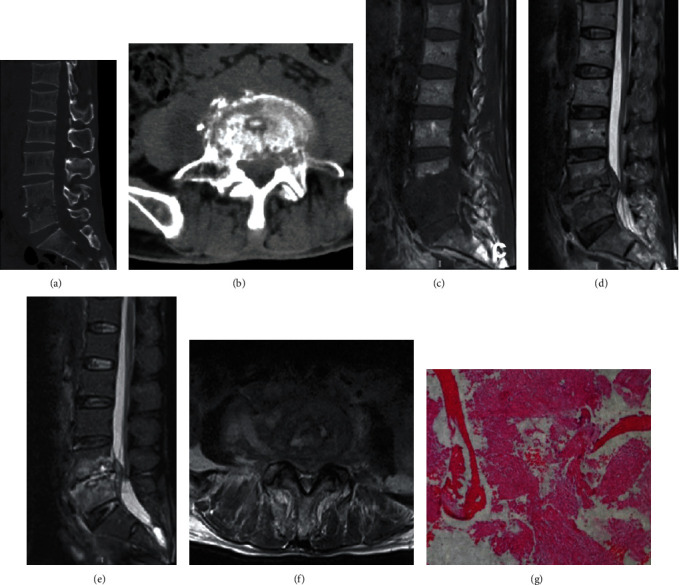
A 56-year-old male suffered from back pain for 4 months with no malignant tumor history. (a, b) lumbar CT showed L4 and L5 vertebral body bone destruction, narrow intervertebral disc, and sequestra formation. (c–f) Lumbar MRI indicated that L4 and L5 vertebral body destruction, subligamentous spread of abscess, and intervertebral disc were involved. (g) The score was 0 points and the preoperative diagnosis was spinal TB which was consistent with the postoperative pathological diagnosis (granulomatous inflammation).

**Figure 6:**
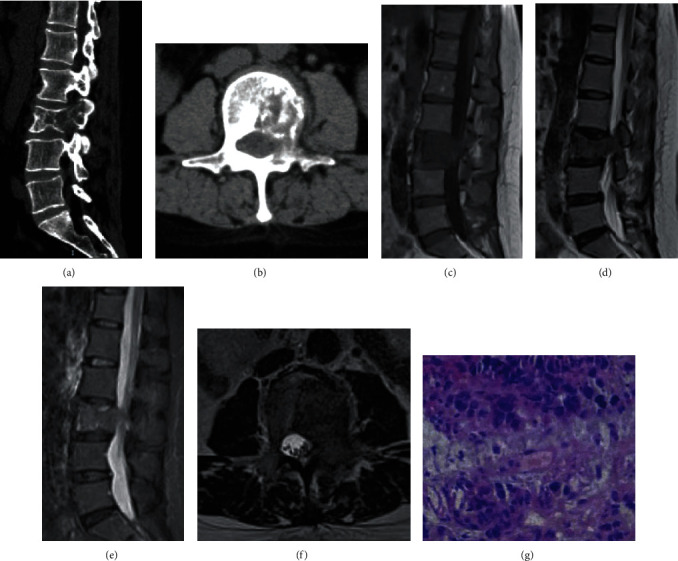
A 42-year-old female suffered from back pain for 6 months and double lower limbs weakness for 10 days with a breast cancer history. (a, b) Lumbar CT showed L3 vertebral body and attachment bone destruction, no subligamentous spread of abscess, no obvious narrow intervertebral space, and sequestra formation. (c–f) Lumbar MRI indicated that L3 vertebral body and attachment bone destruction, but intervertebral disc was normal. (g) The score was 10 points and the preoperative diagnosis was spinal metastasis which was consistent with the postoperative pathological diagnosis (metastatic adenocarcinoma).

Typical cases are shown in Figures 5 and 6.

## 4. Discussion

### 4.1. Clinical Findings

Previous study suggested that the onset age of spinal metastasis was higher than that of spinal TB. But in our study, age was found no contribution to the differential diagnosis, this may due to the expansion of the overlap of the onset age of two diseases [2]. A male predominance in spinal metastasis was also reported [5], but no significant difference in gender was found, and this may because more breast cancer than prostate cancer was included in our study (13 and 10 cases, respectively). Sciubba et al. [20] found that 90% of malignant tumor patients had spinal metastasis, and about 30% of them were admitted to the hospital because of symptomatic spinal metastases. And our study also found that malignant tumor history is an important predictor for spinal metastasis. Momjian et al. reported that more than 50% of spinal TB patients could not find evidence of lung TB [21], which may be why we can hardly found the pulmonary TB history of suspected spinal TB patients at the outpatient department. Thus, in this study, we did not include the TB history for analyzing.

### 4.2. Laboratory Test

Both ESR and CRP were thought helpful for the differential diagnosis of spinal metastasis and spinal TB [10]. However, in our study, the diagnostic specificity of neither ESR nor CRP was satisfactory. We think this may be related to the anti-TB drug treatment before outpatient [22]. All patients included in this study were admitted to our hospital for surgical treatment and most of them had received at least 2 weeks of anti-TB chemotherapy before they went for outpatient, so ESR and CRP might be not high or even normal.

### 4.3. Imaging Examination

Because mycobacterium TB lacks the proteolytic enzymes and cannot destroy the ligaments [23], so once a spinal TB begins as a destructive lesion in one of the anterior margins of the body of a vertebra, it will spread under the anterior longitudinal ligament. Subligamentous spread of a TB abscess under the anterior longitudinal ligament seems to be a unique feature of spinal TB with strong diagnostic accuracy. Jain et al. found that 92% of spinal TB patients had subligamentous spread of abscess [24]. In Kanna et al.'s study, 85% of spinal TB patients had subligamentous spread [25], which is similar with our results (88.67%).

Spinal TB mainly spreads via the anterior vertebral arterial; thus, the TB lesions generally began in the anterior superior border of the vertebra. With the progress of the disease, spinal TB can spread to the posterior part of vertebra causing the infection of the whole vertebra [26]. While spinal metastasis is mainly caused by primary tumor spread through Batson venous plexus [27]. Batson venous plexus match with each other and form a traffic branch on the surface of lamina, process, and articular process [28], and this may be the reason why posterior vertebral elements are involved in most spinal metastases.

Because the mycobacterium TB cannot produce proteolytic enzyme and directly destroy the intervertebral disc structure [23], so in the early stage of spinal TB, intervertebral disc may appear normal or only with mildly signal change [29]. But as a result of that disc nutrition is mainly provided by adjacent vertebra, when both sides of the endplate are destroyed, intervertebral disc loses nutrition supply and thus be damaged [30]. Our study found 8 cases of spinal TB appeared with disc preserved; this may be due to the early stage of the disease. While for spinal metastasis, the intervertebral disc is of poor blood supply, so it is rarely affected [31].

Jain et al. reported four patterns of bone destruction in spinal TB, namely, fragmentary, osteolytic, subperiosteal, and well-defined lytic with sclerotic margins, and the fragmentary type was the most common [32]. Fragmentary type imaged as multiple points and platelet high-density bone in bone destruction zone on CT and they were named sequestra. Sequestra were the bone tissue that loses blood supply, and the growth metabolism was broken off. It is assumed that sequestra formation may be related to that TB inflammatory exudate cannot destroy the bone tissue that loses blood supply due to lack of protein enzyme [23]. While the bone destruction of spinal metastases included osteolytic, osteogenic, and osteolytic-osteogenic mixed [33]. And spinal tumor mainly imaged as osteolytic bone destruction, and sequestra were rare.

Due to the combination of clinical characteristics rather than based on any single feature, there is no doubt that the outpatient scoring system can also improve the accuracy of distinguishing spinal metastasis from atypical spinal TB. For example, Shen et al. [34] reported an atypical spinal TB which was misdiagnosed of spinal metastasis with multilevel and noncontiguous lesions, CT showed that the intervertebral spaces between affected vertebra are narrowed and isolated, small sand-like sequestra can also be seen, and MRI showed that the intervertebral discs were involved, but no paraspinal soft tissues. According to the outpatient scoring system, the score was 1 point, which suggested that the lesion should be spinal TB which was consistent with the pathological diagnosis.

Our study also has limitations. First, certain MRI findings of spinal TB or spinal metastasis can be confused with pyogenic infections and other noninfective disorders, but in this study, we just studied the two with higher incidence (spinal TB and spinal metastasis). Second, the sample size was small and it was impossible to further explore the differential diagnosis of atypical spinal lesions. Third, the clinical symptoms and tumor markers were not analyzed.

## 5. Conclusion

The outpatient scoring system seems to achieve high sensitivity and specificity in distinguishing spinal metastasis from spinal TB. Spinal lesions with the score of 5 to 10 would be considered spinal metastasis, while the score of 0 to 4 is spinal TB. Because the scoring system is mainly based on the clinical characteristics that can be obtained by an outpatient orthopedist, it is suitable to be used as a diagnostic tool in the outpatient department.

## Figures and Tables

**Table 1:** Prevalence and univariate analysis of clinical characteristics of spinal metastasis.

Clinical characteristic	Pathological diagnosis		Sensitivity (%)	Specificity (%)	*P* value
	Spinal metastasis	Spinal TB			
Gender = male	48/82	36/59	58.53	38.98	0.767
Age >50 years	53/82	26/59	64.63	55.93	0.015
Malignant tumor history	29/82	6/53	30.49	88.68	0.004
ESR <65 mm/h	68/82	33/53	82.93	37.74	<0.001
CRP <13 mg/L	54/82	14/53	65.85	73.58	<0.001
No subligamentous spread	72/82	6/53	87.80	88.67	<0.001
No paravertebral or psoas abscess	67/82	11/53	81.70	79.25	<0.001
Isolated or skipped vertebral lesions	68/82	8/59	82.93	86.44	<0.001
Vertebral posterior elements destroyed	61/82	14/59	71.76	76.27	<0.001
Preserved intervertebral discs	74/82	8/59	90.24	86.44	<0.001
No sequestra formation	74/82	23/59	90.24	60.02	<0.001

**Table 2:** Multivariate analysis of clinical characteristics of spinal metastasis.

Clinical characteristics	Regression coefficient (*β*)	*P* value	Odds ratio (OR)
Malignant tumor history	2.362	0.007	10.615
No subligamentous spread	1.617	0.029	5.040
Vertebral posterior elements destroyed	2.199	0.004	9.018
Preserved intervertebral discs	2.779	0.001	16.102
No sequestra formation	2.183	0.014	8.871

**Table 3:** The outpatient scoring system for distinguishing spinal metastasis from spinal TB.

Predictors	Points
Malignant tumor history	
Yes	2
No	0
No subligamentous spread	
Yes	1
No	0
Vertebral posterior elements destroyed	
Yes	2
No	0
Preserved intervertebral disc	
Yes	3
No	0
No sequestra formation	
Yes	2
No	0

**Table 4:** Comparison of performance of the outpatient scoring system on derivation set and validation set.

		Derivation set			Validation set		
		Spinal metastasis	Spinal TB	Total	Spinal metastasis	Spinal TB	Total
Pathological diagnosis	Spinal metastasis	80	2	82	91	2	93
	Spinal TB	8	51	59	7	53	60
	Total	88	53	141	98	55	153

Sensitivity (%)		97.56			97.85		
Specificity (%)		86.44			88.33		

## Data Availability

The clinical data in this study is available from the corresponding author on reasonable request.

## Abbreviations

TB: Tuberculosis
ESR: Erythrocyte sedimentation rate
CRP: C-reactive protein
CT: Computed tomography
MRI: Magnetic resonance imaging
ROC: Receiver operating characteristic
AUC: Area under curve.
